# Grad-CAM Enhanced Explainable Deep Learning for Multi-Class Lung Cancer Classification Using DE-SAMNet Model

**DOI:** 10.3390/diagnostics16050757

**Published:** 2026-03-03

**Authors:** Murat Kılıç, Merve Bıyıklı, Abdulkadir Yelman, Hüseyin Fırat, Hüseyin Üzen, İpek Balikçi Çiçek, Abdulkadir Şengür

**Affiliations:** 1 Turgut Ozal Medical Center, Department of Thoracic Surgery, Faculty of Medicine, Inonu University, Malatya 44280, Türkiye; murat.kilic@inonu.edu.tr (M.K.); merve.biyikli@inonu.edu.tr (M.B.); 2 Department of Computer Technologies, Genç Vocational School, Bingöl University, Bingöl 12000, Türkiye; ayelman@bingol.edu.tr; 3 Department of Computer Engineering, Faculty of Engineering, Dicle University, Diyarbakır 21280, Türkiye; 4 Department of Computer Engineering, Faculty of Engineering and Architecture, Bingöl University, Bingöl 12000, Türkiye; huzen@bingol.edu.tr; 5 Department of Biostatistics and Medical Informatics, Faculty of Medicine, Inonu University Turgut Ozal Medical Center, Malatya 44280, Türkiye; ipek.balikci@inonu.edu.tr; 6 Department of Electrical and Electronics Engineering, Faculty of Technology, Fırat University, Elazığ 23119, Türkiye; ksengur@firat.edu.tr

**Keywords:** lung cancer, classification, densenet121, efficientnetb0, spatial attention module

## Abstract

**Background/Objectives:** Lung cancer (LC) is the leading cause of cancer-related mortality worldwide, making early and accurate diagnosis crucial for improving patient outcomes. Although chest computed tomography (CT) enables detailed assessment of lung abnormalities, manual interpretation is time-consuming, requires expert expertise, and is prone to diagnostic variability. To address these challenges, this study proposes DE-SAMNet, a hybrid deep learning framework for automated multi-class LC classification from CT scans. **Methods:** The model integrates two pre-trained convolutional neural networks—DenseNet121 and EfficientNetB0—operating in parallel to extract complementary multi-scale features. A Spatial Attention Module (SAM) is applied to each feature stream to emphasize clinically important regions. Final classification is performed through a compact fusion mechanism involving global average pooling, batch normalization, and a fully connected layer. DE-SAMNet was evaluated on two datasets: a public dataset (IQ-OTH/NCCD) with benign, malignant, and normal cases, and a private clinical dataset including benign, malignant, cystic, and healthy cases. **Results:** On the public dataset, the model achieved a 99.00% F1-score, 98.41% recall, 99.64% precision, and 99.54% accuracy. On the private dataset, it obtained 95.96% accuracy, 95.99% precision, 96.04% F1-score, and 96.21% recall, outperforming existing approaches. To enhance reliability, explainable AI (XAI) techniques such as Grad-CAM were used to visualize the model’s decision rationale. The resulting heatmaps effectively highlight lesion-specific regions, offering transparency and supporting clinical interpretability. **Conclusions:** This explainability strengthens trust in automated predictions and demonstrates the clinical potential of the proposed system. Overall, DE-SAMNet delivers a highly accurate and interpretable solution for early LC detection.

## 1. Introduction

Lung cancer (LC) is a prevalent and life-threatening disease, recognized as the primary cause of cancer-related mortality globally [1,2]. It affects millions of people each year and is commonly observed in both men and women. This disease is characterized by the formation of malignant tumors that begin in the respiratory tract and often spread rapidly [3,4]. Lung tumors appear as small tissue formations in the form of circular, white shadows on computed tomography (CT) scans [1]. These tumors are classified as either malignant, cystic, or benign. Unlike malignant tumors, which are cancerous and can metastasize to other areas, benign tumors are harmless and do not invade surrounding tissues [1,5,6].

Benign lung tumors are typically slow-growing lesions with a good prognosis and no potential for local invasion or distant metastasis. The most common example within this group is a pulmonary hamartoma. Clinically, the majority of patients are asymptomatic at the time of diagnosis, and the lesions are often detected incidentally as radiological findings. CT is the primary imaging modality in differential diagnosis, and the presence of intralesional fat components or popcorn-like calcifications on imaging carries high predictive value in favor of hamartoma. However, in cases with atypical radiological presentation, histopathological confirmation is required to exclude malignancy [7]. Surgical excision is necessary in symptomatic cases or when malignancy cannot be ruled out. In current surgical practice, video-assisted thoracoscopic surgery (VATS) is widely accepted as the gold-standard minimally invasive approach compared with thoracotomy due to reduced morbidity, shorter hospital stay, and postoperative advantages [8].

Malignant lung tumors, particularly non-small cell lung cancers (NSCLCs), are aggressive neoplasms characterized by rapid growth and the potential for local invasion and distant metastasis. In recent years, early screening programs, molecular genetic testing (EGFR, ALK, ROS1, KRAS, BRAF, HER2, etc.), targeted therapies, and advancements in immunotherapy have fundamentally transformed treatment approaches. Current guidelines emphasize the necessity of comprehensive molecular profiling at the time of diagnosis and highlight the importance of personalized treatment strategies [9].

Cystic lung tumors are not a specific diagnosis but rather a radiological description. These lesions may be associated with benign causes, such as bronchogenic cysts or infection, but can also represent atypical manifestations of lung cancer presenting with cystic air spaces. On tomographic imaging, lesions demonstrating morphological changes such as asymmetric wall thickening or size progression carry an increased risk of malignancy. Therefore, close radiological follow-up and, when necessary, histopathological confirmation are crucial in suspicious cystic lesions [10].

Since LC is often diagnosed at advanced stages, treatment options become limited, and survival rates decrease. Therefore, early diagnosis of the disease can significantly improve patient survival rates by positively influencing the treatment process [2,3,4]. Chest CT scans have emerged as a valuable tool for the early diagnosis of LC [2]. CT provides critical information for the evaluation of lung tumors by enabling the precise detection of abnormal features [11]. However, manual analysis of CT scans is a complex and time-consuming task that requires years of clinical experience [4,5]. This creates a significant workload for specialist doctors and radiologists and may lead to potential misdiagnoses or long waiting times for patients [4]. The onset of LC may present with patterns that are not easily detectable by the human eye, leading to the possibility of overlooking vital information [2]. To overcome these challenges, interest in developing computer algorithms has increased. In particular, artificial intelligence (AI) and deep learning (DL) techniques hold great promise in the diagnosis of LC. DL algorithms can process large volumes of data more quickly and can outperform radiologists and specialist doctors. These techniques can analyze medical images automatically, thereby accelerating the diagnostic process and improving its accuracy [1,5].

### 1.1. Literature Review

In recent years, DL has made significant advancements in the classification and detection of LC within the field of medical imaging. Some of the key studies and their achievements in this area are as follows:

Rana et al. [1] suggested a stacked ensemble approach that integrates four models (VGG19 + CNN, Inception v3 + CNN, EfficientNetB7 + CNN, and Xception + CNN) for classifying LC from CT images. These models were fine-tuned using transfer learning. Experiments conducted on the IQOTH/NCCD dataset achieved 96.89% accuracy (ACC), F1-score (F1), and recall (REC), along with a precision (PRE) of 97.04%.

The DeepNodule-Detect model, proposed by Abe et al. [2], is a powerful DL model designed to automate the diagnosis of LC from patient chest CT scans. This model is an ensemble learning system composed of three different CNNs. Trained on the IQ-OTH/NCCD dataset, it utilizes data augmentation and oversampling of the benign class to address class imbalance issues. Additionally, morphological operations and cropping algorithms are employed for the segmentation of lung regions of interest (ROI). DeepNodule-Detect achieves a specificity of 98.13%, sensitivity of 98.21%, and ACC of 98.17% when categorizing scans as non-cancerous or cancerous. Similarly, when classifying scans into malignant, normal, and benign pulmonary nodules, it attains 95.43% ACC, 93.40% sensitivity, and 97.09% specificity. The results demonstrate superior performance compared to individual CNN models.

Venkatraman and Reddy [12] suggested an approach that integrates DL-based feature extraction with traditional machine learning methods for LC classification. In their study, deep features obtained from a pre-trained VGG16 model were transferred to a separate support vector machine classifier. After filtering out corrupted and duplicate images, the model was trained on the IQOTH/NCCD dataset, which was enriched with normalization and data augmentation techniques. The results showed an ACC of 89.36%, PRE of 90.10%, F1 of 92%, and REC of 91.78%.

In the study by Sabzalian et al. [13], the authors proposed a novel Bidirectional Recurrent Neural Network optimized using an Ebola Search Optimization Algorithm for the classification of LC. This enhanced model benefits from a metaheuristic optimization approach that integrates opposition-based learning to overcome common issues like slow convergence and premature local optima found in traditional optimization methods. The authors validated their method using the IQ-OTH/NCCD LC dataset. The improved method achieved superior performance when preprocessing steps such as noise removal (median filtering), contrast enhancement, and data normalization were applied. On the IQ-OTH/NCCD dataset, the model achieved an F1 of 97.32%, a specificity of 96.48%, a REC of 96.15%, a PRE of 98.52%, and an ACC of 97.06%.

Chaohua Yan et al. [14] proposed a novel approach for LC classification by utilizing a CNN optimized with the Snake Optimization Algorithm. This enhanced optimizer was specifically designed to precisely tune CNN hyperparameters such as the number of convolutional layers, kernel sizes, pooling sizes, and dropout rates, with the aim of improving LC detection accuracy from CT images. The effectiveness of this approach was evaluated using the IQ-OTH/NCCD dataset. After applying preprocessing techniques like brightness normalization, median filtering, and contrast enhancement, the proposed approach achieved an ACC of 96.58%, a sensitivity of 95.38%, and an F1 of 91.53%.

Deepika et al. [15] present a two-stage transfer learning model optimized for LC classification. The method first employs a U-Net model to reduce noise and reconstruct anatomical features in CT images, then uses a ShuffleNet architecture to classify the disease stage on the reconstructed images. The particle swarm optimization technique has been integrated to optimize the model’s hyperparameters. The study also uses the IQ-OTH/NCCD dataset and achieves an ACC of 97.85%.

Raza et al. [16] developed the Lung-EffNet model, based on EfficientNetB1 and utilizing transfer learning, for LC classification. The model was trained on the IQ-OTH/NCCD dataset, and various data augmentation techniques were applied to address class imbalance in the dataset. The Lung-EffNet model achieved an ACC rate of 99.10%.

Ghosh et al. [17] introduced a DL-model approach that integrates a self-attention mechanism into a pre-trained VGG16 architecture to improve lung cancer classification performance from CT images. Their method leverages the representational strength of convolutional neural networks while incorporating transformer-based scaled dot-product attention to better identify clinically relevant image regions. By enhancing the model’s ability to capture global contextual relationships and subtle visual patterns, the proposed framework achieved highly competitive results on the IQ-OTH/NCCD dataset, reporting an average ACC of 97.96% (with a peak of 98.64%) and strong F1, REC, and PRE values (98.10%, 97.96%, and 98.25%).

### 1.2. Literature Gaps

Although recent advancements in DL-based LC classification have demonstrated remarkable progress in tumor detection and classification, several notable limitations remain in the current literature:

• Most of the studies primarily focus on binary classification tasks, typically aiming to distinguish between malignant and benign tumors. The clinical significance of cystic tumors remains underexplored. Cystic lung tumors are often overlooked or grouped together with non-cancerous classes, which limits the level of detail and diagnostic relevance of the results. This is a significant limitation, as cystic lung lesions may present morphological features that overlap with those of malignancies, and inadequate classification models may lead to misinterpretation or delayed diagnosis. Consequently, there is a clear need for multi-class models that explicitly address cystic tumor differentiation.
• Deep learning success strongly depends on large, diverse, and accurately labeled datasets. However, available LC datasets are generally restricted to malignant and benign classes and do not offer cystic tumor samples. This absence prevents robust model training, comparative benchmarking, and reproducibility across cystic lung tumor analysis.
• Although recent deep learning-based lung cancer studies have demonstrated strong diagnostic performance, many of them evaluate their models using a single dataset. This raises concerns regarding generalizability, as results obtained from a single source may be influenced by dataset-specific distributions, imaging characteristics, or class compositions. To address this limitation, the present study evaluates the proposed model on two independent datasets—one public and one private—providing a more rigorous assessment of robustness and clinical applicability.

### 1.3. Innovations and Contributions

In this study, a hybrid DL-based model is proposed for LC classification using chest CT images. The proposed model, called DE-SAMNet, consists of DenseNet121, EfficientNetB0, and Spatial Attention Modules (SAM).

To evaluate the effectiveness of the DE-SAMNet model, two datasets were used: one public dataset (IQ-OTH/NCCD), which includes benign, normal, and malignant classes, and one private dataset containing malignant, benign, cystic, and healthy classes. Experimental results on these datasets show that the DE-SAMNet model achieved an accuracy of 99.54%, precision of 99.64%, recall of 98.41%, and F1-score of 99% on the public IQ-OTH/NCCD dataset, and an accuracy of 95.96%, precision of 95.99%, recall of 96.21%, and F1-score of 96.04% on the private dataset. A comparison with other models from the literature clearly demonstrates the effectiveness of the proposed model. Furthermore, this study introduces several methodological and scientific innovations that directly address the gaps identified in the existing literature.

• A novel ensemble architecture is proposed by integrating two pre-trained convolutional neural networks—DenseNet121 and EfficientNetB0—which are used in parallel to extract complementary low-, mid-, and high-level features. This dual-backbone strategy combines the deep feature reuse capabilities of DenseNet121 with the compound-scaled efficiency of EfficientNetB0, resulting in a robust and informative feature representation.
• A Spatial Attention Module (SAM) is applied individually to the outputs of both DenseNet121 and EfficientNetB0 to enable the network to focus more effectively on clinically relevant spatial regions within lung CT images. This adaptive attention mechanism selectively amplifies important features such as lesions or tumors, thus improving the model’s discriminative capacity without significantly increasing computational complexity.
• This study introduces a newly developed, clinically verified four-class dataset that includes benign, malignant, cystic, and healthy lung tissues, addressing a previously understudied diagnostic area by incorporating cystic lesions. Unlike many existing approaches that are limited to binary classification, the proposed model is capable of effectively distinguishing among these four clinically meaningful classes, providing fine-grained diagnostic information that supports earlier and more accurate decision-making, and enabling radiologists to deliver more tailored and timely interventions.
• By concatenating the attention-enhanced outputs from DenseNet121 and EfficientNetB0, the model captures both low-level and high-level features across different spatial resolutions. This multi-scale feature fusion significantly boosts the model’s ability to distinguish subtle differences in lung tissue appearance, leading to improved performance in detecting early-stage malignancies and differentiating between pathological and healthy structures.
• By training and testing on both public (IQ-OTH/NCCD) and private datasets, this study demonstrates strong model robustness and real-world applicability—addressing the common limitations of dataset dependency and lack of external validation.
• The experimental results demonstrate that the proposed architecture surpasses several leading LC classification models in terms of accuracy, precision, recall, and F1-score. These findings underscore the value of combining ensemble feature extraction with adaptive spatial attention and highlight the potential of the proposed framework to advance automated LC diagnosis.

The rest of the paper is organized as follows: In Section 2, the proposed model and the methods used to create our DE-SAMNet model (DenseNet121, EfficientNetB0, and SAM) are detailed. In Section 3, the public and private datasets used in the study are explained. Additionally, the hyperparameters used in the experimental studies, the evaluation criteria, and the experimental results are described. Finally, Section 4 presents the conclusions, which provide an overall analysis of the study.

## 2. Proposed Model

**Figure 1:**
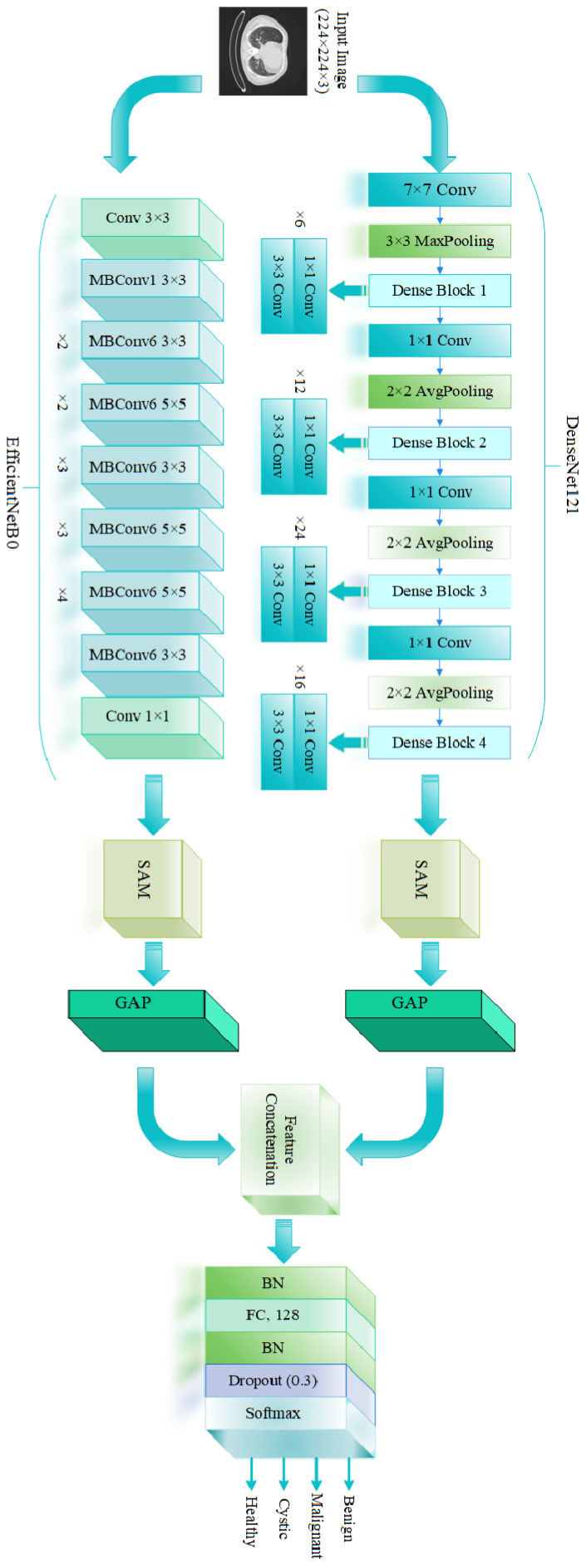
Visualization of the proposed DE-SAMNet model. SAM: Spatial Attention Module, GAP: global average pooling, BN: batch normalization, and FC: fully connected layer.

This study introduces a DL-based hybrid architecture designed for the automatic classification of lung CT scans into four categories: malignant, cystic, normal/healthy, and benign. An overview of the model’s architecture is presented in Figure 1.

The model accepts a CT image input of size 224 × 224 × 3, which is simultaneously processed by two pre-trained convolutional neural networks: DenseNet121 and EfficientNetB0. These networks are employed to extract diverse and complementary feature representations from the input images. DenseNet121 is known for its efficient feature reuse and strong gradient flow, which is achieved through dense connectivity between layers. This architecture helps capture low-level and mid-level visual features critical for identifying subtle patterns in lung lesions. EfficientNetB0 provides an optimized trade-off between model accuracy and computational efficiency through compound scaling. Its lightweight nature and powerful feature extraction capabilities make it suitable for processing high-resolution CT images with minimal loss of relevant information. To further enhance the discriminative power of the extracted features, a Spatial Attention Module (SAM) is applied to the outputs of both DenseNet121 and EfficientNetB0. The SAM selectively emphasizes important spatial regions of the feature maps, allowing the model to focus more effectively on clinically significant areas in the lung, such as nodules, masses, or abnormal tissue regions. Following the application of SAM, a global average pooling (GAP) layer is used to decrease the spatial dimensions and convert the attention-refined feature maps into compact feature vectors. The outputs from both branches are then concatenated to form a unified and rich feature representation. The concatenated features pass through a batch normalization (BN) layer to stabilize and accelerate training. This is followed by a fully connected (FC) layer with 128 units, another BN layer, and a dropout layer (dropout rate = 0.3) to prevent overfitting. Finally, the output layer uses a softmax activation function to classify the input image into one of the four target categories: malignant, cystic, normal/healthy, and benign. The fusion of DenseNet121 and EfficientNetB0 with spatial attention allows the model to capture multi-scale, context-aware, and spatially focused features, which significantly improves classification performance. This ensemble strategy leverages the individual strengths of each component, resulting in a more robust and accurate diagnostic tool for lung disease assessment from CT images.

### 2.1. DenseNet121

DenseNet121 is a CNN model that introduces dense connectivity between layers, where each layer receives input from all preceding layers. This approach enables the reuse of features and strengthens gradient flow during training. In contrast to traditional CNNs, where outputs are passed sequentially from one layer to the next, DenseNet121 concatenates the feature maps from all previous layers and passes them to subsequent layers. This dense connectivity pattern not only improves learning efficiency but also reduces the number of parameters required [18]. In the proposed model, DenseNet121 is utilized as one of the core feature extractors due to its efficient and deep feature reuse capability. DenseNet121 begins with a 7 × 7 convolutional layer followed by max pooling and proceeds through four dense blocks, each separated by transition layers. Each dense block consists of multiple convolutional layers (1 × 1 followed by 3 × 3), where each layer receives inputs from all preceding layers within the block, enabling rich feature reuse and strengthened gradient flow. The transition layers include 1 × 1 convolutions and 2 × 2 average pooling operations to reduce the spatial dimensions while preserving relevant features [18]. At the end of the final dense block, GAP is applied before the features are passed to the classifier. In the context of our DE-SAMNet model, DenseNet121 is employed to extract low-level and mid-level features from the lung CT images. These features are crucial for detecting subtle patterns associated with early-stage lung abnormalities. The dense connections help preserve spatial details and ensure the effective propagation of relevant features, making DenseNet121 an ideal choice for medical image analysis tasks. In our model, the output from the GAP layer of DenseNet121 is further enhanced using SAM, which helps the network focus on critical lung regions. These refined features are then concatenated with those from EfficientNetB0 to form a comprehensive representation used for classifying CT images into benign, malignant, and normal categories.

### 2.2. EfficientNetB0

EfficientNetB0 is the baseline model of the EfficientNet family, known for its optimal balance between model accuracy and computational efficiency. It employs a compound scaling method that uniformly scales depth, width, and resolution using a set of carefully selected coefficients [19]. This design enables EfficientNetB0 to achieve high performance with significantly fewer parameters and lower computational cost compared to traditional CNN architectures. The architecture begins with a standard 3 × 3 convolutional layer followed by a series of mobile inverted bottleneck (MBConv) blocks, which are the core building units of the model. Each MBConv block includes depthwise separable convolutions, squeeze-and-excitation modules, and skip connections, which together enhance computational efficiency and model capacity. EfficientNetB0 consists of seven stages of MBConv blocks with varying expansion factors, filter sizes, and strides, allowing the model to capture both fine-grained and high-level semantic features. The final layers include a 1 × 1 convolution, followed by GAP, and an FC layer with a softmax activation for classification [19]. This streamlined architecture makes EfficientNetB0 highly suitable for medical image analysis tasks where accuracy and efficiency are both critical. In our model, EfficientNetB0 serves as a complementary backbone alongside DenseNet121. While DenseNet121 excels at preserving fine-grained features, EfficientNetB0 focuses on extracting high-level semantic representations from the CT images. This combination provides a richer and more diverse feature set, which is beneficial for accurately distinguishing between malignant, cystic, normal, and benign lung tissues.

### 2.3. Spatial Attention Module

**Figure 2:**
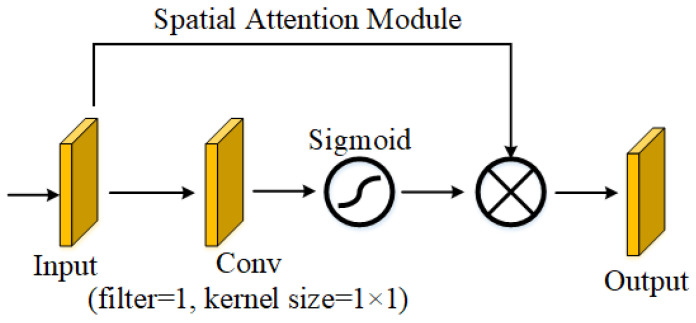
Structure of SAM [22].

In our DE-SAMNet model, the Spatial Attention Module (SAM) significantly improves feature representation by enabling the network to concentrate on the most relevant spatial areas within the input CT images. As illustrated in Figure 2, SAM takes a feature map as input and applies a 1 × 1 convolutional operation with a single filter. This operation is designed to learn a spatial attention map, which identifies which spatial regions should be emphasized or suppressed. The resulting output is passed through a sigmoid activation function, which scales the values between 0 and 1, effectively creating a soft attention mask. This attention mask is then element-wise multiplied with the original input feature map, amplifying relevant regions (such as lesions or tumors) and attenuating less informative areas (e.g., background). This refined feature map is then passed on to the next stage of the network. Importantly, the module is lightweight and introduces minimal computational overhead, making it well-suited for deep learning applications in medical imaging [20,21,22]. In the proposed model, SAM is applied to the outputs of both DenseNet121 and EfficientNetB0, enabling the network to adaptively focus on spatially significant regions extracted by each backbone. This attention-driven enhancement improves the discriminative power of the combined features, which is particularly beneficial for distinguishing between malignant, cystic, normal, and benign lung tissues in CT images. Integrating SAM enhances DE-SAMNet’s resilience to irrelevant variations and improves its focus on clinically significant structures.

## 3. Experimental Results

### 3.1. Datasets

In this research, two separate LC datasets were utilized. The initial dataset is the publicly available IQ-OTH/NCCD. This dataset was acquired over a 3-month period during the fall of 2019 at the Iraq-Oncology Teaching Hospital/National Center for Cancer Diseases. It comprises CT scans from patients diagnosed with LC at various stages, as well as from healthy individuals. Overall, the dataset contains 1097 images, representing slices from CT scans of 110 cases. These cases are categorized into three groups: malignant, benign, and normal. Specifically, there are 55 normal, 15 benign, and 40 malignant cases. The total number of CT images in each group is as follows: 416 normal, 120 benign, and 561 malignant [23].

**Figure 3:**
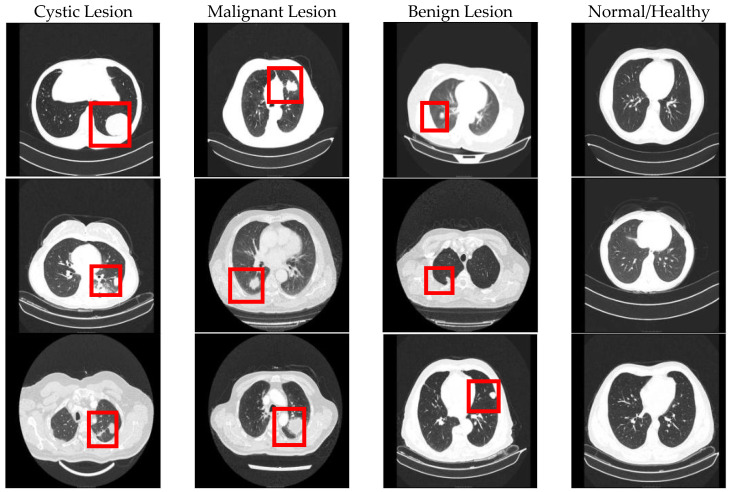
Example CT images in the datasets.

The CT images used in the second dataset (our private dataset) were collected from cases who applied to the Department of Thoracic Surgery at Turgut Ozal Medical Center, Inonu University. The dataset contains a total of 3843 CT images from 273 cases. These cases are divided into four groups: 70 malignant, 63 benign, 70 cystic, and 70 healthy. The total number of CT images in each group is as follows: 1000 healthy, 1000 cystic, 1000 malignant, and 843 benign. The data in the dataset were supported within the scope of the TÜBİTAK 1001 program and conducted as part of the research project numbered 125E062. The data were collected with the approval of the Inonu University Scientific Research and Publication Ethics Committee under permission number [2024/6289]. Additionally, no data augmentation techniques were applied to either dataset used in this study. All experimental analyses were conducted using only the original CT images obtained from the publicly available IQ-OTH/NCCD dataset and the private dataset collected at Turgut Ozal Medical Center, Inonu University. Thus, the models were trained and evaluated exclusively on real, unmodified data without applying any artificial transformations. Sample CT images from both datasets are shown in Figure 3.

### 3.2. Setting of Hyperparameters

This study was conducted using Python 3.12.12, with all code executed on Kaggle Notebooks. To accelerate the model training process, a P100 GPU was employed. The deep learning model was trained using carefully chosen hyperparameters. CT images resized to 224 × 224 × 3 dimensions served as input. The Adam optimizer was utilized for model optimization, set with a learning rate of 0.0001. Given the multi-class classification nature of the task, sparse categorical cross-entropy was selected as the loss function. The training process was carried out over 100 epochs with a batch size of 64. Notably, no data augmentation techniques were applied to the datasets during the experiments.

Two complementary experimental settings were employed to ensure a comprehensive and reliable evaluation of the proposed model. In the first setting, both the public and private datasets were randomly divided into 80% for training and 20% for testing in order to obtain baseline performance results and enable direct comparison with existing studies in the literature, where fixed hold-out validation is commonly used. Note that data partitioning was performed in an image-based manner; however, to prevent potential data leakage, CT slices belonging to the same patient were not included in both the training and testing sets. During data partitioning, all slices belonging to a single patient were kept within only one subset (training or testing). However, performance estimates derived from a single train–test split may be sensitive to data partitioning, particularly in medical imaging applications with limited sample sizes and potential class imbalance. To address this limitation and enhance the robustness and generalizability of the reported results, a 5-fold cross-validation strategy was additionally conducted as an independent evaluation protocol. In this setup, the model was iteratively trained and tested on different subsets of the data, with 80% of the samples used for training and the remaining 20% for testing in each fold. This approach reduces the risk of biased performance estimation and provides a more reliable assessment of the model’s stability across varying data distributions. Consequently, the combined use of both evaluation strategies enables fair comparison with prior work while ensuring a statistically more robust and reliable performance analysis.

### 3.3. Evaluation Criteria

To evaluate the effectiveness of the proposed model, four key metrics were utilized: accuracy (ACC), precision (PRE), recall (REC), and F1-score (F1).

The ACC indicates the overall rate of correct predictions made by the model, offering a broad measure of its performance.

The PRE reflects the proportion of true positives among all instances labeled as positive, highlighting the model’s ability to minimize false positives.

The REC, also known as sensitivity, measures the proportion of actual positive cases correctly detected, showing the model’s effectiveness in identifying relevant instances.

The F1, calculated as the harmonic mean of PRE and REC, provides a balanced evaluation, especially useful when dealing with imbalanced datasets or when both types of errors—false negatives and false positives—carry significance. Collectively, these metrics deliver a thorough assessment of the classification results.

All metrics are calculated according to Equations (1)–(4) below.

(1) $$\mathrm{ACC}=\frac{\mathrm{TP}+\mathrm{TN}}{\mathrm{TP}+\mathrm{TN}+\mathrm{FP}+\mathrm{FN}}$$

(2) $$\mathrm{PRE}=\frac{\mathrm{TP}}{\mathrm{TP}+\mathrm{FP}}$$

(3) $$\mathrm{REC}=\frac{\mathrm{TP}}{\mathrm{TP}+\mathrm{FN}}$$

(4) $$F1=2\times \frac{\mathrm{PRE}\times \mathrm{REC}}{\mathrm{PRE}+\mathrm{REC}}$$

In the above equations, the terms TP, TN, FP, and FN are derived from the confusion matrix: True positives (TP) refer to cases where the model correctly identifies the presence of LC in a CT image. True negatives (TN) denote cases where the model accurately detects the absence of LC. False positives (FP) arise when the model incorrectly predicts LC in an image without any malignancy, which could lead to unnecessary tests or patient anxiety. False negatives (FN) occur when the model fails to detect an existing case of LC, potentially delaying essential treatment. Together, these metrics form a robust evaluation framework to thoroughly assess the clinical applicability of the proposed model in detecting LC from CT scans.

### 3.4. Results

#### 3.4.1. Results of Public Dataset (IQ-OTH/NCCD)

Table 1 presents a comprehensive comparison of the proposed DE-SAMNet model with various state-of-the-art DL architectures, including DenseNet121 [18], DenseNet201 [18], EfficientNetB0 [19], ConvNeXtTiny [24], InceptionV3 [25], MobileNet [26], ResNet50 [27], VGG16 [28], and Xception [29]. The models were evaluated using four standard performance metrics: ACC, PRE, REC, and F1.

The proposed DE-SAMNet model achieves the highest overall classification performance among all compared architectures. It reaches an ACC of 99.54%, PRE of 99.64%, REC of 98.41%, and F1 of 99.00%, outperforming all baseline models across every evaluation metric. This indicates that DE-SAMNet not only makes highly accurate predictions but also maintains a strong balance between PRE and REC, resulting in superior overall reliability.

When compared with strong competitors such as DenseNet121 and EfficientNetB0, DE-SAMNet still demonstrates a clear advantage. DenseNet121 achieves relatively high performance (98.63% ACC and 97.12% F1), and EfficientNetB0 also shows competitive results with lower computational complexity. However, both models remain below DE-SAMNet in all performance metrics, showing that the proposed model improves predictive capability while preserving stable classification behavior.

Compared to deeper or computationally heavier networks such as DenseNet201, InceptionV3, and Xception, DE-SAMNet achieves significantly better classification results while avoiding excessive computational cost. Although these architectures generally provide robust feature extraction, their accuracy and F1-scores remain around the mid-90% range, demonstrating that DE-SAMNet offers a more effective feature representation and decision-making capability.

Against lightweight architectures such as MobileNet, DE-SAMNet provides a substantial improvement in overall performance. MobileNet has a relatively low parameter count and computational demand, but its REC and F1 are considerably lower, suggesting reduced robustness. DE-SAMNet achieves much higher predictive performance while still maintaining moderate model complexity, indicating a good balance between efficiency and effectiveness.

In comparison with more traditional architectures such as ResNet50, VGG16, and ConvNeXtTiny, the superiority of DE-SAMNet becomes more evident. These models show lower accuracy and F1-scores and, in some cases, require higher computational resources (e.g., VGG16 with very high GFLOPs). DE-SAMNet improves both predictive performance and computational efficiency relative to these models, highlighting its advantage as a practical and high-performing solution.

From a complexity perspective, DE-SAMNet uses 11.3 M parameters and 6.5 GFLOPs, which places it in a moderate range. While it is not the smallest model, it is considerably lighter than models like VGG16, ResNet50, and ConvNeXtTiny, yet delivers superior performance. This demonstrates that the proposed architecture achieves an effective trade-off between accuracy and computational cost, making it suitable for practical deep learning applications.

In summary, the results clearly demonstrate that DE-SAMNet not only surpasses individual state-of-the-art models but also offers a more balanced and highly accurate solution for the classification task. This significant improvement is due to the integration of multiple model strengths and potentially the use of attention mechanisms or hybrid feature fusion strategies within DE-SAMNet.

Table 2 presents a comparative analysis of studies conducted in the field of lung cancer classification using the publicly available IQ-OTH/NCCD dataset, along with the performance of the proposed DE-SAMNet model. The comparison is based on four key metrics: ACC, PRE, REC, and F1. These metrics are crucial for evaluating the overall effectiveness of classification models.

An examination of the reported performance values in the literature shows a wide range of results, with our proposed model outperforming many existing approaches.

Firstly, in terms of ACC, the study by Venkatraman & Reddy (2024) [12] reported the lowest performance with 89.36%, while other works achieved values ranging between 96% and 99%. The highest accuracy rate of 99.10% was reported by Raza et al. (2023) [16]. However, the proposed DE-SAMNet model achieved an even higher accuracy of 99.54%, surpassing all previous studies and demonstrating its superior ability to correctly classify lung cancer images in the dataset.

Regarding the PRE metric, most previous studies achieved results between 96% and 99%. Specifically, Güraksın et al. (2025) [5] reported 99.06%, Gupta et al. (2025) [30] reported 98.70%, Raza et al. (2023) [16] reported 99.10%, and Ma et al. (2024) [31] reported 99.45%. DE-SAMNet outperformed all of these with a PRE rate of 99.64%, minimizing false positives and improving the model’s prediction reliability. This outcome indicates that the vast majority of instances predicted as positive by our model were correctly classified.

For the REC, Raza et al. (2023) [16] achieved the highest score of 99.12%, while the DE-SAMNet model obtained a slightly lower value of 98.41%. Although Raza et al. (2023) [16] reported a marginally higher REC, DE-SAMNet compensates for this with its superior PRE and ACC. High REC is particularly important in detecting critical diseases like cancer, as it helps reduce the rate of false negatives. The strong REC performance of DE-SAMNet indicates that the likelihood of missing cancerous cases remains low.

The F1 provides a balanced evaluation of both PRE and REC. In the reviewed literature, this metric ranged from 91.53% (Yan and Razmjoooy, 2023 [14]) to 99.08% (Raza et al., 2023 [16]). The DE-SAMNet model achieved an F1 of 99.00%, ranking second-highest among all studies and demonstrating robust overall performance. Thanks to its high ACC and PRE, our model shows great potential as a reliable decision support tool in clinical applications. Overall, Table 2 highlights that the proposed DE-SAMNet model outperformed most existing approaches in lung cancer classification. It achieved the best results for ACC and PRE while also ranking highly for REC and F1. These findings suggest that DE-SAMNet is capable of more effective feature extraction and accurate classification on the public IQ-OTH/NCCD dataset, making it a strong candidate for future AI-based clinical decision support systems aimed at lung cancer diagnosis.

**Figure 4:**
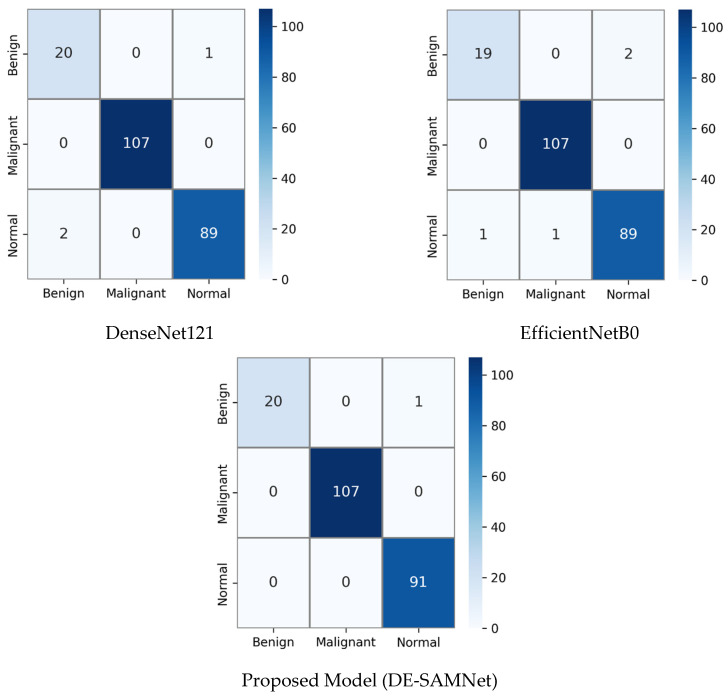
Confusion matrices of the proposed DE-SAMNet model and its backbone networks, DenseNet121 and EfficientNetB0 for public dataset (80% of the data used for training, 20% for testing).

The confusion matrices in Figure 4 provide a clear comparative analysis of the classification performance of DenseNet121, EfficientNetB0, and DE-SAMNet across three classes—benign, malignant, and normal—using a public lung dataset.

In the DenseNet121 model, the confusion matrix reveals a high ACC classification overall. All 107 malignant cases were correctly identified without any misclassifications. Among the benign samples, 20 were classified correctly, while one sample was mistakenly identified as normal. For the normal class, 89 out of 91 instances were correctly classified, but two were incorrectly predicted as benign. These results indicate that while DenseNet121 is highly accurate for malignant detection, it shows a slight tendency to confuse benign and normal categories.

The EfficientNetB0 model also demonstrates strong performance, particularly for the malignant class, where it, like DenseNet121, achieved a perfect classification rate with 107 correct predictions. For benign samples, 19 were accurately classified, while two were misclassified as normal. The normal class had 89 correctly identified instances, with one sample misclassified as benign. Compared to DenseNet121, EfficientNetB0 shows a similar level of accuracy in the malignant class but a slightly higher rate of error in the benign class, which suggests marginally reduced robustness in distinguishing benign from normal cases.

In contrast, the proposed model (DE-SAMNet), which integrates DenseNet121 and EfficientNetB0 with attention mechanisms, outperforms the individual backbone networks. It correctly classified all 107 malignant and all 91 normal cases, demonstrating perfect accuracy for these two classes. For the benign class, 20 out of 21 cases were correctly predicted, with only a single instance misclassified as normal. This results in only one misclassification across all three classes, indicating superior discriminative capability and generalization performance.

In summary, while DenseNet121 and EfficientNetB0 both show excellent performance in identifying malignant cases, their accuracy drops slightly when distinguishing between benign and normal categories. The DE-SAMNet, however, effectively minimizes this confusion and achieves nearly flawless classification performance, demonstrating the advantage of the ensemble and attention-based approach in enhancing feature representation and decision-making accuracy.

Table 3 presents a comparative evaluation of the proposed DE-SAMNet model and various pre-trained CNN architectures using four key metrics—ACC, PRE, REC, and F1—obtained through five-fold cross-validation.

The results indicate that all widely used and powerful architectures, including ConvNeXtTiny, DenseNet121/201, EfficientNetB0, InceptionV3, MobileNet, ResNet50, VGG16, and Xception, achieve accuracy values above 96%, demonstrating both the learnability of the problem and the generally stable performance of the evaluated models. Nevertheless, the proposed DE-SAMNet outperforms all competing methods across all metrics, achieving 98.08% ACC and REC, 98.04% PRE, and a 97.99% F1-score. The improvement observed, particularly in the F1-score, suggests that the model effectively balances false positives and false negatives, leading to more consistent class discrimination. Although architectures such as DenseNet121 and EfficientNetB0 exhibit relatively high performance, the simultaneous superiority of DE-SAMNet across all metrics indicates that the discriminative feature extraction and attention mechanisms incorporated into the proposed architecture directly contribute to its classification performance. Moreover, the use of a five-fold cross-validation strategy confirms that these results are not limited to a single data split but instead reflect a robust and generalizable performance across different subsets, supporting the reliability of the proposed model for clinical or real-world applications.

**Figure 5:**
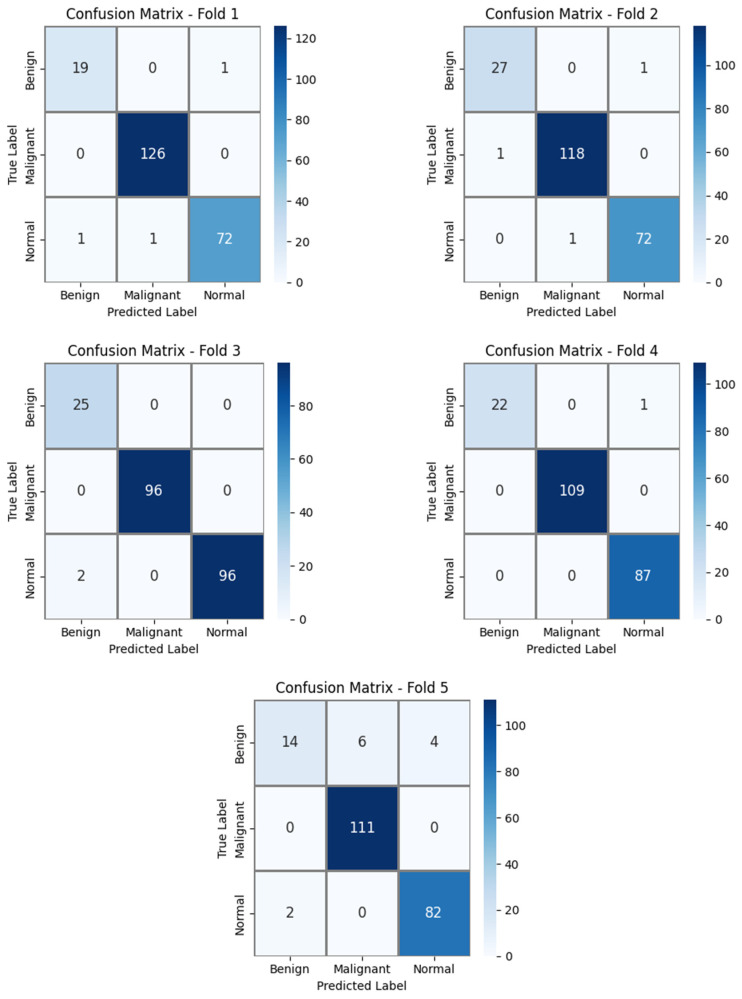
Confusion matrices for each fold obtained using 5-fold cross-validation with the proposed DE-SAMNet model for public dataset.

The confusion matrices corresponding to the five different folds presented in Figure 5 provide a detailed illustration of the prediction consistency and error distribution of the proposed DE-SAMNet model throughout the five-fold cross-validation process.

The clear dominance of the diagonal elements across all folds indicates that the model can accurately discriminate between benign, malignant, and normal classes. In particular, the consistently high number of correct predictions for the malignant class, along with the near absence of misclassifications across all folds, demonstrates the model’s high sensitivity toward this clinically critical category. Although limited mutual confusions between the benign and normal classes are observed in all folds, the number of such errors remains low and does not significantly affect the overall performance. The nearly error-free classification achieved in Fold 3 and Fold 4 (with only two normal samples misclassified as benign in Fold 3 and only one benign sample misclassified as normal in Fold 4) indicates that the model can produce highly robust and stable results for certain data splits. Despite the relatively higher number of errors observed in the benign class in Fold 5 (with six samples misclassified as malignant and four as normal), the preservation of high accuracy for the malignant and normal classes suggests that the model is generally resilient to class imbalance or variations in sample distribution.

Overall, these confusion matrices confirm that DE-SAMNet delivers consistent, balanced, and generalizable classification performance across different data subsets, producing reliable predictions, especially for clinically critical classes.

#### 3.4.2. Results of Our Private Dataset

Table 4 presents a comparative performance evaluation between DE-SAMNet and a set of widely used pre-trained CNN models—ConvNeXtTiny, DenseNet121-201, EfficientNetB0, InceptionV3, MobileNet, ResNet50, VGG16, and Xception—using a private lung dataset. The comparison is based on four evaluation metrics: ACC, PRE, REC, and F1. These metrics are critical in assessing the model’s overall predictive performance, especially in medical imaging applications where both PRE and REC are vital.

The proposed DE-SAMNet clearly outperforms all other models across every metric. It achieves the highest ACC of 95.96%, significantly ahead of the next best-performing model, DenseNet201 with 93.53%, and DenseNet121 with 92.70%.

In terms of PRE, which reflects the model’s ability to correctly identify positive cases, DE-SAMNet scores 95.99%, again outperforming all other models. The closest in PRE are DenseNet201 (93.61%) and DenseNet121 (92.70%).

When considering REC, which is crucial for ensuring that actual positive cases are not missed, DE-SAMNet achieves 96.21%, surpassing DenseNet201 (93.77%) and DenseNet121 (92.87%). This high REC is particularly important in clinical settings where false negatives can have serious consequences.

The F1, which balances PRE and REC, is highest for DE-SAMNet at 96.04%, indicating its robustness in both identifying positive cases and minimizing false positives. This is notably better than the F1 of DenseNet201 (93.66%), DenseNet121 (92.67%), and ConvNeXtTiny (91.62%). Models like Xception and VGG16 show relatively lower performance across all metrics, with ACC values of 87.36% and 88.67%, respectively, and correspondingly lower PRE, REC, and F1. This suggests that while these models may have been effective in general-purpose image classification tasks, they might lack the specialized representational power needed for lung disease classification tasks.

Table 4 also provides a comparison of the proposed DE-SAMNet with several widely used pre-trained CNN models in terms of model complexity and computational cost, represented by the number of parameters (Params) and floating-point operations (GFLOPs). These two indicators are essential for evaluating the practical feasibility of deep learning models, especially in medical imaging applications where computational efficiency and deployment constraints are important considerations.

From the perspective of model size, DE-SAMNet contains 11.3 M parameters, indicating a moderate architectural complexity. It is significantly lighter than larger models such as ConvNeXtTiny (27.9 M), ResNet50 (23.8 M), InceptionV3 (22.07 M), and Xception (21.1 M), demonstrating that the proposed model avoids excessive parameter growth. Although some lightweight models, such as EfficientNetB0 (4.2 M), MobileNet (3.3 M), and DenseNet121 (7.1 M), have fewer parameters, DE-SAMNet maintains a balanced model size while achieving stronger overall performance.

In terms of computational cost, DE-SAMNet requires 6.5 GFLOPs, which places it within a moderate range compared to other architectures. It is computationally more efficient than heavy models such as VGG16 (30.7 GFLOPs), Xception (9.1 GFLOPs), ConvNeXtTiny (8.7 GFLOPs), and ResNet50 (7.7 GFLOPs). While certain lightweight architectures, including EfficientNetB0 (0.8 GFLOPs) and MobileNet (1.1 GFLOPs), demand fewer operations, these models typically prioritize efficiency at the expense of predictive power.

Overall, the results indicate that DE-SAMNet achieves a favorable trade-off between model complexity and computational efficiency. Its moderate parameter size and GFLOPs suggest that it is computationally practical while avoiding the heavy resource requirements observed in several conventional deep CNN architectures, making it suitable for efficient deployment in real-world clinical environments.

In summary, DE-SAMNet demonstrates superior generalization and diagnostic capabilities on the private lung dataset compared to all baseline pre-trained CNNs. Its strong performance across all evaluation metrics highlights its effectiveness and reliability for clinical decision support in lung disease classification.

**Figure 6:**
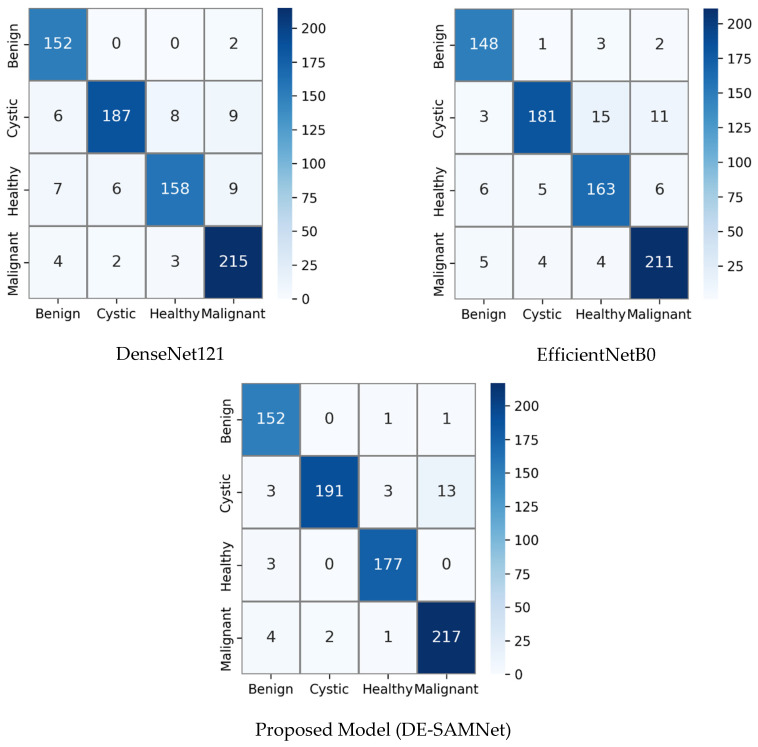
Confusion matrices of the proposed DE-SAMNet model and its backbone networks, DenseNet121 and EfficientNetB0 for our private dataset (80% of the data used for training, 20% for testing).

Figure 6 presents the confusion matrices for DenseNet121, EfficientNetB0, and the proposed DE-SAMNet model using our private dataset. A comparative analysis of these results reveals important insights regarding the classification performance of each model across the four classes: benign, cystic, healthy, and malignant.

DenseNet121 demonstrates strong overall classification performance, particularly for the malignant class, with 215 correct predictions and very few misclassifications (a total of nine misclassifications). Similarly, the cystic class shows high accuracy with 187 correct predictions, though it misclassifies a few images as healthy (eight) and malignant (nine). For the benign class, 152 images are correctly classified, with only minor misclassifications into the malignant class (two). The healthy class sees 158 correct predictions but has some confusion with the cystic, benign, and malignant classes. Overall, DenseNet121 exhibits solid performance, but with some noticeable misclassifications, especially between cystic and other disease types.

EfficientNetB0, in comparison, shows slightly lower performance. It correctly classifies 211 malignant cases (four fewer than DenseNet121) but exhibits more misclassifications into the other classes. The cystic class is also more affected here, with only 181 correct classifications and 29 misclassified as other categories, particularly healthy (15) and malignant (11). The healthy class sees 163 correct predictions, slightly higher than DenseNet121, but the benign class suffers from a few more errors, with 148 correctly predicted cases. Overall, EfficientNetB0 tends to confuse benign and cystic with malignant more frequently than DenseNet121, indicating a slight drop in sensitivity for these three categories.

The proposed DE-SAMNet model outperforms both backbone networks in nearly all categories. For the benign class, it achieves perfect consistency with DenseNet121, correctly classifying 152 samples and misclassifying only two. The cystic class sees a notable improvement, with 191 correct classifications—higher than both DenseNet121 (187) and EfficientNetB0 (181). Despite minor confusion between healthy and malignant, this is the best performance among the three. The healthy class benefits significantly from DE-SAMNet, with 177 correct predictions and only three misclassifications, improving over both backbones. Lastly, the malignant class achieves the highest correct classification (217), again outperforming the two backbone models. These results suggest that the integration of spatial attention in DE-SAMNet helps the model focus more effectively on discriminative features, reducing inter-class confusion.

In summary, while both DenseNet121 and EfficientNetB0 offer competent classification capabilities, the proposed DE-SAMNet achieves superior performance across all categories. This confirms that the spatial attention mechanism enhances the model’s discriminative ability and robustness, especially in challenging cases such as distinguishing between cystic and malignant types.

The results obtained on the private dataset using 5-fold cross-validation are presented in Table 5.

The comparative analysis demonstrates that the proposed DE-SAMNet model, which incorporates a spatial attention mechanism, significantly outperforms both its closest competitor, ResNet50, and the other pre-trained CNN architectures across all evaluation metrics. DE-SAMNet achieves 92.32% ACC, 92.35% PRE, 92.32% REC, and a 92.30% F1, providing a consistent improvement of approximately 0.4–0.5% over ResNet50 in all metrics. This performance gain can be attributed to the spatial attention mechanism, which enhances class discrimination by focusing on the spatially salient tumor regions while suppressing background interference. Although architectures such as DenseNet121/201, VGG16, and MobileNet yield competitive results, they fail to reach the performance level of DE-SAMNet in terms of both F1-score and overall accuracy. ConvNeXtTiny exhibits moderate performance, whereas the relatively lower metric values of InceptionV3 and Xception indicate that these models are less effective in capturing the complex spatial characteristics of the private dataset.

Overall, DE-SAMNet demonstrates a balanced and superior performance across all metrics, highlighting that its spatial attention-based design enables more focused feature extraction and leads to a more reliable and generalizable classification performance.

**Figure 7:**
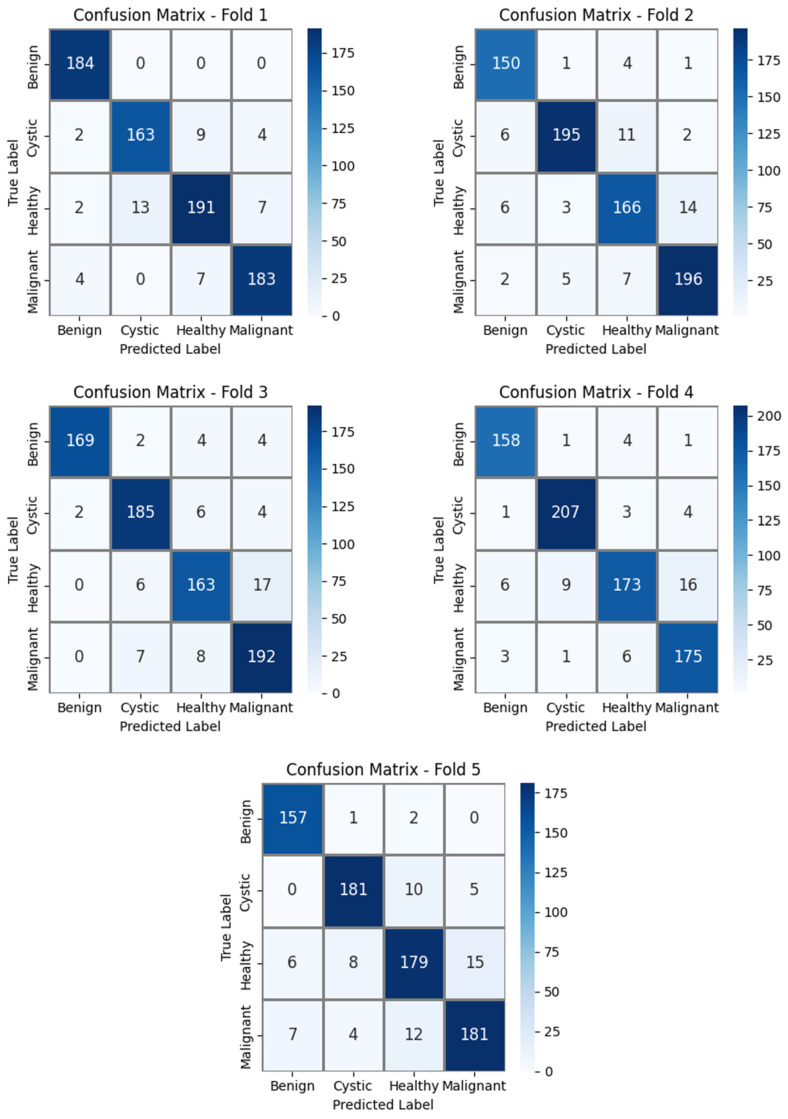
Confusion matrices for each fold obtained using 5-fold cross-validation with the proposed DE-SAMNet model for our private dataset.

In the experimental studies conducted on the private dataset using 5-fold cross-validation, the confusion matrices obtained for each fold of the proposed DE-SAMNet model are presented in Figure 7. In Fold 1, the benign class is classified without any errors, while misclassifications are mainly concentrated among the cystic, healthy, and malignant classes. The highest number of misclassifications in this fold occurs when the healthy class is predicted as cystic. In Fold 2, high correct classification rates are maintained for all classes; however, an increase in errors is observed where the healthy class is predicted as malignant, and the cystic class is predicted as healthy. A similar trend continues in Fold 3, where high accuracy is achieved, particularly for the malignant class, while the healthy class exhibits relatively more misclassifications compared to the other classes, mainly being predicted as malignant. Fold 4 stands out as one of the folds with the highest overall performance, exhibiting one of the strongest diagonal distributions across all classes. This indicates that the model possesses a stable generalization capability across different data splits. In this fold as well, the most frequent misclassification occurs when the healthy class is predicted as malignant. In Fold 5, a performance consistent with the previous folds is observed; however, limited mutual confusion between the healthy and malignant classes persists.

Overall, the minor performance variations among the folds stem from the natural variability in data distribution, and the presence of similar error patterns across all folds clearly demonstrates that DE-SAMNet provides a stable, balanced, and reliable classification performance.

**Figure 8:**
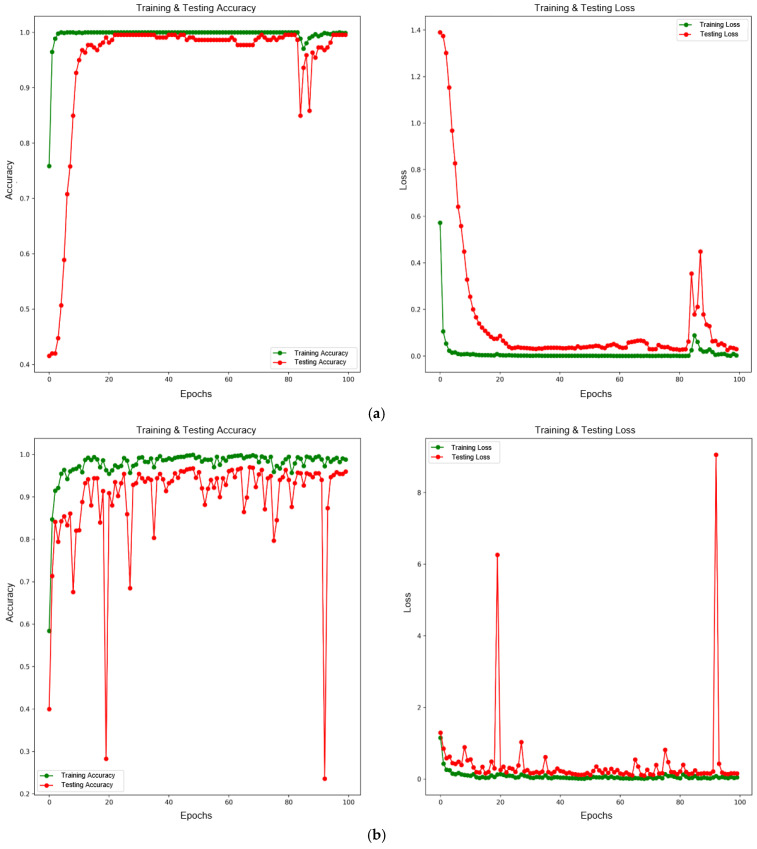
Accuracy–loss curves of the proposed DE-SAMNet model. (**a**) Public IQ-OTH/NCCD dataset. (**b**) Our private dataset.

The accuracy–loss curves obtained for the proposed model in both the public IQ-OTH/NCCD dataset and the private dataset are shown in Figure 8.

In Figure 8a, which presents the results for the public IQ-OTH/NCCD dataset, the training accuracy rapidly increases during the initial epochs and quickly reaches nearly 100%, while the testing accuracy also rises sharply and stabilizes around 98–100% throughout most of the training process. This indicates that the model effectively captures the underlying patterns in the data and generalizes well to unseen samples. The training loss decreases steeply and approaches near zero after approximately 15 epochs, while the testing loss also converges to a low level with only minor fluctuations. Although slight spikes in testing loss and drops in testing accuracy are observed around later epochs (e.g., after epoch 80), these variations are not severe, and the overall performance remains consistently high, confirming that the model achieves robust and reliable results on the public dataset.

Figure 8b presents the accuracy–loss curves of the proposed model when applied to our private dataset, and the results reveal a notable degree of instability compared to the public dataset. The training accuracy curve shows a steady upward trend, stabilizing above 95% after the initial epochs, indicating that the model effectively learns the underlying patterns within the training data. Similarly, the training loss decreases gradually and remains at a consistently low level after approximately 20 epochs, confirming that the model fits the training data well.

However, the testing accuracy curve exhibits significant fluctuations throughout the 100 training epochs. Although testing accuracy frequently aligns with training accuracy at high levels, several sharp drops are observed, in some cases falling below 50% or even 30%. These abrupt declines highlight that the model struggles to generalize consistently to unseen samples, reflecting variability and potential complexity in the private dataset. A similar pattern is evident in the loss curves: while training loss remains stable, the testing loss demonstrates pronounced spikes at certain epochs (e.g., around epochs 20 and 90), occasionally exceeding a value of 8. These spikes directly correspond to the abrupt declines in testing accuracy, indicating substantial misclassification during those specific evaluation phases.

In our current implementation, a fixed learning rate was used throughout training, and no learning rate scheduler or early stopping mechanism was employed. The model was trained for a fixed 100 epochs, and the reported results correspond to the epoch achieving the best testing (validation) accuracy rather than the final epoch. Importantly, the instability observed in the testing curves occurs despite the stability of the training curves, suggesting that the issue is not optimization divergence or uncontrolled overfitting.

Instead, the fluctuations appear to be associated with sensitivity to specific test-set distributions at certain epochs. Given the relatively limited size of the private dataset and the batch size of 64, a small number of misclassified samples—particularly from minority classes—can disproportionately influence epoch-level metrics. Since evaluation is conducted at the slice level, misclassification of several challenging slices within a batch may significantly increase cross-entropy loss values, resulting in visible spikes in testing loss and corresponding drops in testing accuracy. Greater heterogeneity and potential class imbalance within the private dataset further amplify this variance.

Importantly, the instability is episodic rather than progressive. The model rapidly recovers in subsequent epochs, and the overall trajectory of the testing loss remains downward across training. Moreover, the 5-fold cross-validation results demonstrate consistent average performance across folds, indicating that the observed fluctuations do not reflect systematic optimization failure or persistent overfitting but rather variance arising from dataset complexity and limited sample size.

Overall, while the generalization performance on the private dataset is less stable than on the public dataset, the combined evidence from training stability, best-epoch model selection, and cross-validation supports the conclusion that the model is able to extract meaningful and discriminative features, albeit with higher sensitivity to data variability in the private cohort.

### 3.5. Grad-CAM Analysis

**Figure 9:**
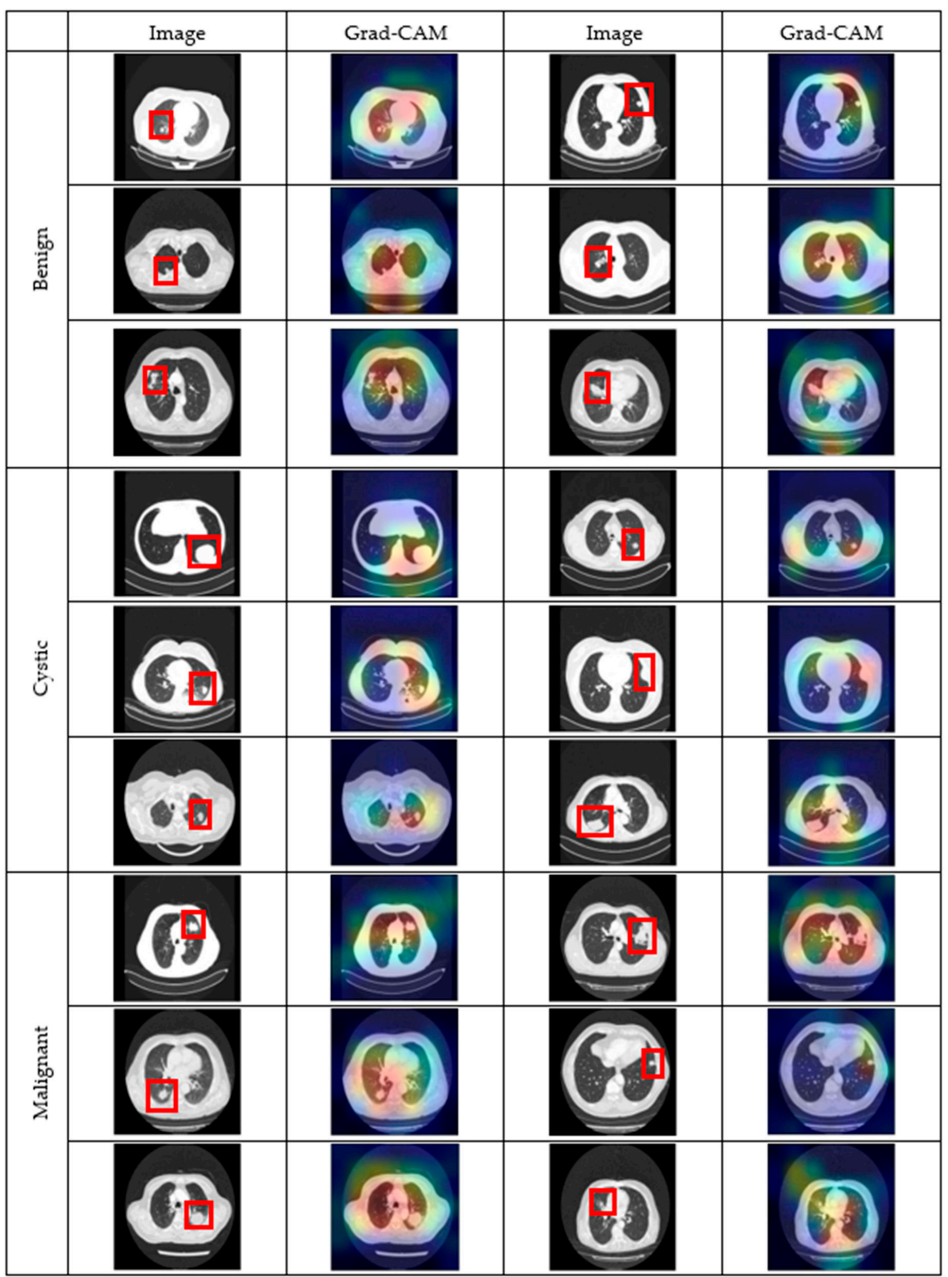
Grad-CAM examples for benign, cystic, and malignant lesions.

In Figure 9, Grad-CAM examples are provided for benign, cystic, and malignant lung lesions.

Lesion-focusing success was evaluated by examining whether the highest activation regions corresponded to radiologically abnormal areas in the CT images. Across all lesion categories, the model predominantly concentrated its attention on lesion-related regions rather than irrelevant background structures, indicating that the classification decisions were largely driven by clinically meaningful image features.

For benign lesions, which commonly present as well-circumscribed nodules or localized opacities, Grad-CAM activations were generally centered on the lesion but occasionally extended toward neighboring lung tissue. This moderate diffusion of attention suggests that the model incorporates contextual cues surrounding the lesion, which is reasonable given the heterogeneous nature and variable appearance of benign findings. Despite this diffusion, the main activation area remained spatially aligned with the pathological region, reflecting satisfactory lesion localization.

Malignant lesions exhibit irregular borders, heterogeneous density, and surrounding parenchymal alterations such as spiculation or infiltration. The Grad-CAM maps for malignant cases frequently show broader activation areas covering both the lesion core and its peripheral margins. This wider focus indicates that the model captures not only the central tumor mass but also adjacent tissue changes that are clinically associated with malignancy. While the activation boundaries may appear less sharply defined compared to those of cystic lesions, the attention remains anatomically plausible and reflects the invasive growth patterns commonly observed in malignant lung tumors.

By contrast, the cystic lesions—particularly hydatid cysts—exhibit distinct imaging characteristics that make them more readily identifiable. Hydatid cysts appear as round, well-defined, low-density structures, often with smooth borders and relatively homogeneous internal contents. These features are strongly captured in the Grad-CAM maps, where the model consistently highlights the cystic cavity with precise localization. Unlike benign and malignant examples, there is little extraneous activation outside the lesion, demonstrating that the model has learned the defining morphological features of hydatid cysts. This sharper and more consistent attention explains why the proposed model achieves higher success in detecting cystic lesions compared to other categories.

From a clinical perspective, the Grad-CAM results demonstrate that the proposed model bases its predictions on anatomically relevant regions rather than non-diagnostic image patterns. The broader attention observed in malignant cases aligns with radiological practice, where surrounding parenchymal changes and lesion margins play a critical role in malignancy assessment. Similarly, precise activation within cystic cavities supports the clinical validity of the model’s decision-making process.

Importantly, the visual explanations provided by Grad-CAM improve transparency and support human–AI collaboration. By allowing clinicians to verify whether model attention overlaps with expected lesion areas, Grad-CAM facilitates trust, aids diagnostic validation, and reduces concerns related to black-box behavior. Therefore, the interpretability analysis strengthens the clinical applicability of the proposed framework as a decision-support system rather than a purely predictive model.

Overall, the comparison of Grad-CAM results across lesion types shows that while benign and malignant lesions present challenges due to their variability and overlapping radiological features, cystic (hydatid) lesions are more consistently identified. The model’s ability to focus accurately on the cystic cavities without misdirected attention confirms its strength in this domain. This interpretability not only validates the diagnostic performance of the model but also provides clinicians with visual evidence that enhances trust in the automated system.

### 3.6. Ablation Analysis

Table 6 presents the ablation analysis on both the public and private datasets, clearly demonstrating the contribution of the SAM and the effectiveness of the proposed DE-SAMNet architecture.

*Effect of Adding SAM (Public Dataset):* When SAM is integrated into DenseNet121, the accuracy increases from 98.63% to 99.02% (+0.39%), precision from 96.60% to 97.17% (+0.57%), recall from 97.68% to 98.22% (+0.54%), and F1-score from 97.12% to 97.56% (+0.44%). Similarly, adding SAM to EfficientNetB0 improves accuracy from 98.17% to 98.63% (+0.46%) and yields a substantial precision gain from 97.29% to 98.96% (+1.67%). Although recall shows a slight fluctuation (96.09% to 96.46%, +0.37%), the overall F1-score increases from 96.67% to 97.63% (+0.96%). These consistent improvements indicate that SAM enhances feature representation by enabling the model to focus on more discriminative regions.

*Effect of Adding SAM (Private Dataset):* A similar trend is observed on the private dataset. DenseNet121 + SAM improves accuracy from 92.70% to 93.48% (+0.78%), precision from 92.70% to 93.48% (+0.78%), recall from 92.87% to 93.67% (+0.80%), and F1-score from 92.67% to 93.19% (+0.52%). For EfficientNetB0, incorporating SAM increases accuracy from 91.53% to 92.89% (+1.36%), precision from 91.49% to 92.78% (+1.29%), recall from 91.76% to 93.12% (+1.36%), and F1-score from 91.55% to 92.88% (+1.33%). The improvements are even more pronounced on the private dataset, suggesting that SAM contributes to better generalization in more challenging or heterogeneous data distributions.

*Effectiveness of the Proposed DE-SAMNet:* The proposed DE-SAMNet achieves the best performance across all metrics on both datasets. On the public dataset, it attains 99.54% accuracy, 99.64% precision, 98.41% recall, and 99.00% F1-score, outperforming all single-backbone and dual-backbone variants. Compared to the dual-backbone model without the enhanced design (DenseNet121 + EfficientNetB0), DE-SAMNet improves accuracy from 99.08% to 99.54% (+0.46%) and F1-score from 98.66% to 99.00% (+0.34%). On the private dataset, DE-SAMNet further demonstrates its robustness, achieving 95.96% accuracy and a 96.04% F1-score, clearly surpassing all baseline configurations. Notably, compared to the best non-proposed variant (DenseNet121 + EfficientNetB0), it improves accuracy by +1.78% (94.18% → 95.96%) and F1-score by +1.52% (94.52% → 96.04%).

## 4. Discussion

### 4.1. Interpretation of Results

The experimental findings demonstrate that the proposed DE-SAMNet model delivers successful performance in multi-class lung cancer classification, surpassing both its backbone networks and a wide range of state-of-the-art deep learning models. The model achieved 99.54% ACC, 99.64% PRE, 98.41% REC, and 99% F1 on the public dataset, while obtaining 95.96% ACC, 95.99% PRE, 96.21% REC, and 96.04% F1 on our private dataset. These results confirm its applicability to heterogeneous imaging environments. Notably, the system showed particularly strong performance in identifying malignant lesions, which hold critical clinical importance. Confusion matrix outcomes indicate minimal class overlap, highlighting the discriminative capability provided by the dual feature extraction (DenseNet121 and EfficientNetB0) structure and the spatial attention mechanism. The enhanced performance observed in ACC, PRE, REC, and F1 metrics demonstrates that DE-SAMNet effectively captures complex morphological features associated with lung tissue abnormalities, even when imaging characteristics across tumor classes are subtle or overlapping.

### 4.2. Model Robustness

The stability and robustness of the proposed model were rigorously evaluated through fixed train–test splits as well as five-fold cross-validation, performed independently on two separate datasets. Consistency in performance across folds indicates that DE-SAMNet maintains generalizable predictive behavior despite variations in data distribution. In addition, the model’s resilience was demonstrated through sustained performance on a more heterogeneous private dataset that encompasses four clinically relevant classes. Grad-CAM-based visual explainability further verified that the model consistently attends to anatomically plausible lesion regions, substantiating its internal decision logic and reducing the risk of spurious feature reliance. Collectively, these indicators show that the model is both reliable and adaptable across diverse imaging and clinical conditions.

### 4.3. Comparison with Related Work

When compared with existing deep learning approaches reported in the literature, DE-SAMNet demonstrates advantages in both methodological design and classification performance. While many prior studies have focused primarily on binary (benign–malignant) or three-class (benign–malignant–healthy) classification tasks, the proposed framework addresses a more clinically realistic four-class (cystic–benign–malignant–healthy) problem by explicitly incorporating cystic lesions. Quantitatively, DE-SAMNet achieves higher or comparable accuracy and F1-score values than most state-of-the-art models evaluated on the IQ-OTH/NCCD dataset. Methodologically, the dual-backbone architecture leverages complementary feature extraction capabilities, while the spatial attention mechanism enhances focus on diagnostically relevant regions. The enhanced accuracy—combined with strong feature localization and reduced misclassification rates—indicates that the architectural innovations incorporated into DE-SAMNet deliver meaningful advantages over traditional CNN, ensemble, and hybrid models presented in the current literature.

### 4.4. Clinical Implications

The clinical implications of this study are significant. Accurate differentiation among malignant, benign, cystic, and healthy lung tissues can substantially support radiologists in diagnostic decision-making. Reliable identification of malignant lesions may facilitate earlier intervention and improve patient prognosis, while accurate classification of cystic lesions may help avoid unnecessary invasive procedures. Moreover, the explainability provided by Grad-CAM enhances transparency and interpretability, which are critical for clinical acceptance of AI-based systems. As a decision-support tool, DE-SAMNet has the potential to reduce diagnostic workload, improve consistency across readers, and serve as a valuable adjunct to clinical expertise.

### 4.5. Limitations

A primary limitation of this study is that the imaging data were processed using 2D CT slices rather than full 3D volumetric representations. While slice-based analysis enables efficient training and reduces computational complexity, it may limit the model’s ability to capture volumetric context, inter-slice continuity, and the true three-dimensional morphology of lung lesions. Certain spatial characteristics—such as lesion extent, shape continuity, and relationships across adjacent slices—may therefore be underrepresented. Future studies incorporating 3D CNN architectures and DICOM-based volumetric CT data could address this limitation by enabling more comprehensive spatial feature learning and potentially further improving classification performance.

## 5. Conclusions

Lung cancer (LC) continues to be one of the most fatal and prevalent forms of cancer worldwide, underscoring the urgent need for effective early diagnostic solutions. While CT scans have become a vital tool in LC detection, their manual interpretation remains a time-intensive and error-prone process that depends heavily on the experience and attention of radiologists. In this context, DL-based computer-aided diagnosis systems offer a promising alternative by automating the analysis of complex medical images with high accuracy and speed. In this study, we suggested DE-SAMNet, a novel hybrid DL architecture developed to classify lung conditions from CT images into four clinically significant classes: malignant, cystic, benign, and normal. The model capitalizes on the strengths of two powerful CNNs—DenseNet121 and EfficientNetB0—which run in parallel to extract a wide variety of features across multiple scales. By incorporating SAM for each feature stream, the model is able to focus on the most diagnostically relevant areas within the lung images, thereby enhancing its discriminative capabilities.

Experimental evaluations were conducted on both a public benchmark dataset (IQ-OTH/NCCD) and a real-world clinical dataset. The DE-SAMNet model demonstrated superior performance on both, achieving 99.54% ACC on the public dataset and approximately 96% ACC on the private dataset. These results highlight the model’s strong generalization ability and practical utility in clinical environments. One of the standout contributions of this work is its ability to perform fine-grained multi-class classification, which is more aligned with real-world diagnostic needs than binary classification systems. Accurate differentiation among malignant, benign, cystic, and healthy lung tissues can substantially support radiologists in diagnostic decision-making. Reliable identification of malignant lesions may facilitate earlier intervention and potentially improve patient prognosis, while accurate classification of cystic lesions may help avoid unnecessary invasive procedures. Furthermore, the explainability provided through Grad-CAM enhances transparency and interpretability, which are critical factors for the clinical acceptance of AI-based systems. As a decision-support tool, DE-SAMNet has the potential to reduce diagnostic workload, improve consistency among readers, and serve as a valuable adjunct to clinical expertise.

Overall, the proposed DE-SAMNet system demonstrates a substantial enhancement in automated LC identification. Its architecture not only enhances performance but also maintains computational efficiency, making it suitable for potential clinical deployment. This research lays the groundwork for the integration of AI-driven tools in radiology, ultimately contributing to more accessible, consistent, and accurate LC screening practices.

In future studies, it is intended to process CT data directly in its original 3D form using the DICOM format rather than 2D slice-based representations. To better capture the spatial context and volumetric characteristics of lung lesions, 3D CNN-based architectures are planned to be employed, which may further improve clinical applicability and diagnostic robustness.

## Figures and Tables

**Table 1:** Performance analysis of DE-SAMNet and pre-trained CNNs for public dataset (80% of the data used for training, 20% for testing).

Model	ACC (%)	PRE (%)	REC (%)	F1 (%)	Params (M)	GFLOPs
ConvNeXtTiny	95.89	94.05	91.93	92.91	27.9	8.7
DenseNet121	98.63	96.60	97.68	97.12	7.1	5.7
DenseNet201	96.80	93.58	92.66	93.09	18.5	8.6
EfficientNetB0	98.17	97.29	96.09	96.67	4.2	0.8
InceptionV3	96.35	93.25	92.30	92.75	22.07	5.6
MobileNet	97.26	97.94	91.75	94.28	3.3	1.1
ResNet50	95.43	93.53	90.29	91.74	23.8	7.7
VGG16	95.43	92.14	93.95	92.96	14.7	30.7
Xception	96.35	97.33	92.19	94.41	21.1	9.1
DE-SAMNet	99.54	99.64	98.41	99.00	11.3	6.5

**Table 2:** Evaluating the DE-SAMNet against prior studies in the literature.

Author (Year)	ACC (%)	PRE (%)	REC (%)	F1 (%)	Train–Test Split
Rana et al. (2025) [1]	96.89	97.04	96.89	96.89	70-15-15
Abe et al. (2025) [2]	98.17	-	98.21	-	80-20
Güraksın et al. (2025) [5]	99.00	99.06	98.82	98.94	80-20
Venkatraman & Reddy (2024) [12]	89.36	90.10	91.78	92.00	-
Sabzalian et al. (2023) [13]	97.06	98.52	96.15	97.32	-
Yan and Razmjooy (2023) [14]	96.58	95.38	84.16	91.53	75-25
Deepika, R. et al. (2024) [15]	97.85	96.68	97.68	97.15	-
Raza et al. (2023) [16]	99.10	99.10	99.12	99.08	80-20
Gupta et al. (2025) [30]	96.82	98.70	97.50	98.24	80-20
Ma et al. (2024) [31]	97.32	99.45	98.20	98.82	-
Ghosh et al. (2025) [17]	98.64	98.25	97.96	98.10	80-20
Proposed DE-SAMNet model	99.54	99.64	98.41	99.00	80-20
Proposed DE-SAMNet model	98.08	98.04	98.08	97.99	5-fold cross-validation

**Table 3:** Performance analysis of DE-SAMNet and pre-trained CNNs for public dataset (5-fold cross-validation results).

Model	ACC (%)	PRE (%)	REC (%)	F1 (%)	Confidence Intervals Across Folds
ConvNeXtTiny	96.17 ± 1.5	96.25 ± 1.2	96.17 ± 1.5	96.13 ± 1.3	[94.2%, 98.1%]
DenseNet121	97.81 ± 0.6	97.97 ± 0.6	97.81 ± 0.6	97.75 ± 0.6	[97.05%, 98.57%]
DenseNet201	96.90 ± 1.3	96.87 ± 1.4	96.90 ± 1.3	96.81 ± 1.3	[95.28%, 98.52%]
EfficientNetB0	97.54 ± 1.3	97.56 ± 1.3	97.54 ± 1.3	97.48 ± 1.3	[95.91%, 99.17%]
InceptionV3	94.80 ± 0.5	94.65 ± 0.5	94.80 ± 0.5	94.60 ± 0.6	[94.15%, 95.45%]
MobileNet	97.17 ± 1.5	97.22 ± 1.5	97.17 ± 1.5	97.11 ± 1.6	[95.27%, 99.07%]
ResNet50	97.54 ± 1.6	97.60 ± 1.7	97.54 ± 1.6	97.50 ± 1.7	[95.47%, 99.61%]
VGG16	96.90 ± 1.6	96.89 ± 1.7	96.90 ± 1.6	96.86 ± 1.7	[94.90%, 98.91%]
Xception	96.72 ± 1.1	96.71 ± 1.1	96.72 ± 1.1	96.60 ± 1.1	[95.36%, 98.07%]
DE-SAMNet	98.08 ± 2.03	98.04 ± 2.16	98.08 ± 2.03	97.99 ± 2.25	[95.6%, 100%]

**Table 4:** Performance analysis of DE-SAMNet and pre-trained CNNs for our private dataset (80% of the data used for training, 20% for testing).

Model	ACC (%)	PRE (%)	REC (%)	F1 (%)	Params (M)	GFLOPs
ConvNeXtTiny	91.53	91.57	91.74	91.62	27.9	8.7
DenseNet121	92.70	92.70	92.87	92.67	7.1	5.7
DenseNet201	93.53	93.61	93.77	93.66	18.5	8.6
EfficientNetB0	91.53	91.49	91.76	91.55	4.2	0.8
InceptionV3	89.06	88.96	89.34	89.11	22.07	5.6
MobileNet	90.88	90.95	90.89	90.84	3.3	1.1
ResNet50	90.36	90.31	90.67	90.42	23.8	7.7
VGG16	88.67	88.90	88.79	88.82	14.7	30.7
Xception	87.36	87.32	87.34	87.33	21.1	9.1
DE-SAMNet	95.96	95.99	96.21	96.04	11.3	6.5

**Table 5:** Performance analysis of DE-SAMNet and pre-trained CNNs for our private dataset (5-fold cross-validation results).

Model	ACC (%)	PRE (%)	REC (%)	F1 (%)	Confidence Intervals Across Folds
ConvNeXtTiny	88.23 ± 1.8	88.34 ± 1.8	88.23 ± 1.8	88.23 ± 1.8	[86.04%, 90.44%]
DenseNet121	90.45 ± 1.03	90.47 ± 1.04	90.45 ± 1.03	90.45 ± 1.04	[89.18%, 91.72%]
DenseNet201	90.71 ± 0.9	90.73 ± 0.9	90.71 ± 0.9	90.71 ± 0.9	[89.57%, 91.86%]
EfficientNetB0	89.95 ± 0.9	89.97 ± 1.0	89.95 ± 0.9	89.93 ± 1.0	[89.12%, 91.19%]
InceptionV3	82.59 ± 1.5	82.66 ± 1.5	82.59 ± 1.5	82.56 ± 1.5	[80.84%, 84.34%]
MobileNet	90.24 ± 1.7	90.30 ± 1.8	90.24 ± 1.7	90.24 ± 1.7	[88.85%, 91.63%]
ResNet50	91.85 ± 0.8	91.89 ± 0.8	91.85 ± 0.9	91.81 ± 0.8	[90.84%, 92.88%]
VGG16	90.71 ± 0.9	90.74 ± 0.9	90.71 ± 0.9	90.69 ± 0.9	[89.48%, 91.95%]
Xception	87.95 ± 2.7	88.10 ± 2.7	87.95 ± 2.7	87.96 ± 2.7	[84.50%, 91.41%]
DE-SAMNet	92.32 ± 1.08	92.35 ± 1.08	92.32 ± 1.08	92.30 ± 1.09	[91.0%, 93.6%]

**Table 6:** Ablation analysis.

	Public Dataset				Our Private Dataset			
Model	ACC (%)	PRE (%)	REC (%)	F1 (%)	ACC (%)	PRE (%)	REC (%)	F1 (%)
DenseNet121	98.63	96.60	97.68	97.12	92.70	92.70	92.87	92.67
DenseNet121 + SAM	99.02	97.17	98.22	97.56	93.48	93.48	93.67	93.19
EfficientNetB0	98.17	97.29	96.09	96.67	91.53	91.49	91.76	91.55
EfficientNetB0 + SAM	98.63	98.96	96.46	97.63	92.89	92.78	93.12	92.88
DenseNet121+ EfficientNetB0	99.08	99.28	98.10	98.66	94.18	94.24	94.64	94.52
Proposed DE-SAMNet	99.54	99.64	98.41	99.00	95.96	95.99	96.21	96.04

## Data Availability

The original data presented in the study are openly available in https://www.kaggle.com/datasets/hamdallak/the-iqothnccd-lung-cancer-dataset (accessed on 15 December 2025). This study was conducted using Python, and all code was executed on Kaggle Notebooks. Our Python code is open source and will be made available upon request after the publication of the paper.

## Abbreviations

The following abbreviations are used in this manuscript:

LC	Lung cancer
CT	Computed tomography
SAM	Spatial attention module
XAI	Explainable AI
VATS	Video-assisted thoracoscopic surgery
NSCLCs	Non-small cell lung cancers
AI	Artificial intelligence
DL	Deep learning
BN	Batch normalization
GAP	Global average pooling
FC	Fully connected
ACC	Accuracy
PRE	Precision
REC	Recall
F1	F1-score
M	Million
